# Associations Among Estrogens, the Gut Microbiome and Osteoporosis

**DOI:** 10.1007/s11914-024-00896-w

**Published:** 2024-11-25

**Authors:** Miloslav Kverka, Jan J. Stepan

**Affiliations:** 1 Laboratory of Cellular and Molecular Immunology, Institute of Microbiology, Czech Academy of Sciences, Prague, Czechia; 2 Institute of Rheumatology, Prague, Czechia; 3 Department of Rheumatology, First Faculty of Medicine, Charles University, Kateřinská 32, Praha 2, 121 08 Czech Republic

**Keywords:** Osteoporosis, Microbiota, Estrogen, Inflammation, Leaky gut, Ovariectomy, Aging

## Abstract

**Purpose of the Review:**

The purpose of this Review was to summarize the evidence on the associations among estrogen status, cellular senescence, the gut microbiome and osteoporosis.

**Recent Findings:**

Indicate that osteoporosis is a global public health problem that impacts individuals and society. In postmenopausal women, a decrease in estrogen levels is associated with a decrease in gut microbial diversity and richness, as well as increased permeability of the gut barrier, which allows for low-grade inflammation. The direct effects of estrogen status on the association between bone and the gut microbiome were observed in untreated and treated ovariectomized women. In addition to the direct effects of estrogens on bone remodeling, estrogen therapy could reduce the risk of postmenopausal osteoporosis by preventing increased gut epithelial permeability, bacterial translocation and inflammaging. However, in studies comparing the gut microbiota of older women, there were no changes at the phylum level, suggesting that age-related comorbidities may have a greater impact on changes in the gut microbiota than menopausal status does.

**Summary:**

Estrogens modify bone health not only by directly influencing bone remodeling, but also indirectly by influencing the gut microbiota, gut barrier function and the resulting changes in immune system reactivity.

## Introduction

**Fig. 1:**
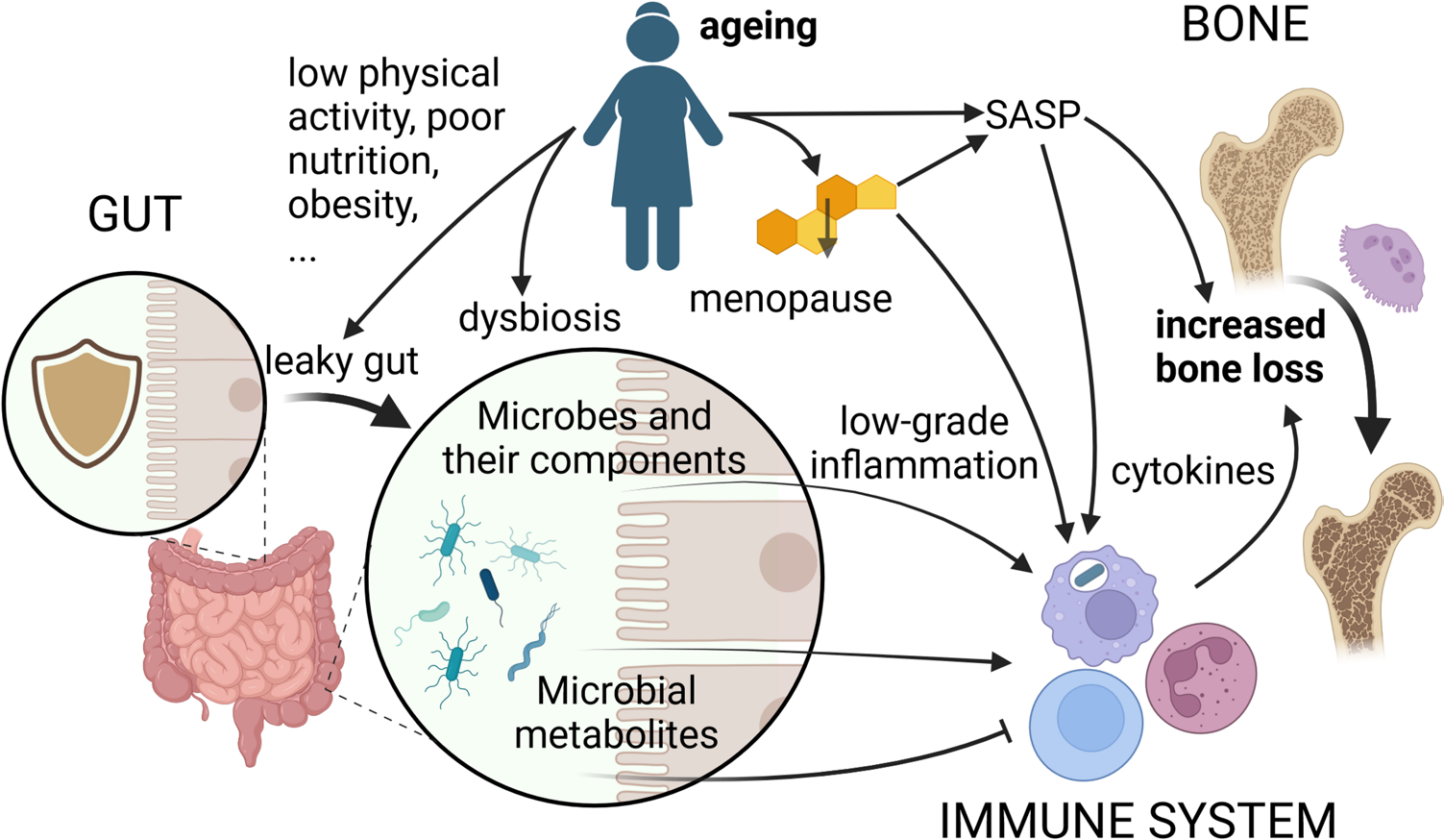
Aging and increasing bone loss are linked via dysbiosis of the intestinal microbiota, a leaky gut, low-grade inflammation and immune system senescence. SASP—senescence-associated secretory phenotype. Created with BioRender.com

Several factors contribute to the development of primary osteoporosis, such as aging, estrogen deficiency, low physical activity, poor nutritional status, changes in immune function, inflammatory conditions, and genetics. Aging is the most important risk factor for osteoporosis and various other age-related diseases. The loss and deterioration of bone mass progress with age from the fourth decade onward [1]. Hormonal changes play an important role in this process. These changes begin many months before the end of the menstrual cycle and can last for several years. The severity and duration of bone and hormonal changes vary from individual to individual and are influenced by genetics and exposure to various factors throughout life (exposome). Given that the population is aging, the consequences of osteoporosis and fractures are increasingly significant medical and economic problems [2]. Most fractures occur in women who do not have osteoporosis according to bone densitometry [3]. Therefore, maintaining a sufficient quantity and quality of bone mass before fractures occur is important. In this review, we aimed to emphasize that the decrease in bone mass and quality in women with increasing age and estrogen deficiency is associated with the persistence of chronic low-grade inflammation in the bones and the gut (Fig. 1). Suppressing this inflammation could be a way to reduce the risk of osteoporosis in clinical practice.

### Bone Aging is Associated with Estrogens

Cellular senescence is an irreversible arrest of the cell cycle that prevents apoptosis and is associated with chronic low-grade inflammation. Senescent cells produce a mixture of proinflammatory cytokines, chemokines and extracellular matrix-degrading proteins, which are referred to as the senescence-associated secretory phenotype (SASP). The SASP induces the aging of neighboring cells in an autocrine and paracrine manner, leading to chronic inflammation and the accumulation of senescent cells [4]. Senescent bone cells accumulate DNA damage, stop proliferating and actively resist apoptosis, thereby impairing their function. With age-associated thymic involution and a decline in bone marrow, the proportion of naive T and B lymphocytes decreases, and the number of autoreactive T cells increases [5–7], which may induce a proinflammatory SASP phenotype [8]. Myeloid cells and senescent osteocytes primarily develop a proinflammatory phenotype in bone [9]. The SASPs contribute to the senescence of immune cells, which share stromal progenitor cells and are subjected to the effects of coregulatory factors (RANKL, TNF-α, and IFN-γ) along with bone cells in the bone marrow. The senescence of bone marrow mesenchymal stem cells and their differentiation into adipocytes, as well as the suppression of bone formation by osteoblasts, are also induced by senescent neutrophils and monocyte-macrophages [10]. Interactions between inflammation-inducing factors and imunosenescence-inducing factors contribute to chronic inflammation; these interactions are characterized by increases in the concentrations of proinflammatory cytokines (e.g., IL-6) and acute-phase proteins [11].

Osteocytes, which are terminally differentiated cells that arise from osteoblasts, play a crucial role in bone aging [12, 13]. With increasing age, bone remodeling and bone microdamage repair are impaired by the loss of osteocytes and changes in the lacunar and canalicular networks of osteocytes [14]. Some SASPs produced by senescent osteocytes induce an increase in osteoclastic bone resorption in a paracrine manner, whereas others trigger the apoptosis of osteoblasts and osteocytes [9, 15]. Wei et al. demonstrated that the level of estrogen is negatively correlated with osteocyte and osteoblast senescence, mainly by regulating p53 protein levels. In mice, accelerated degradation of p53 after ovariectomy suppressed the expression of senescence markers and thus promoted the restoration of bone mass [16]. The removal of senescent osteocytes prevents age-related bone loss by altering the balance of bone remodeling by osteoclasts and osteoblasts [17]. In menopausal women treated with oral conjugated equine estrogen and transdermal 17β-estradiol, the serum levels of several markers of cell senescence are lower than those in women treated with a placebo [18]. Therefore, aging accelerates bone mass loss by reducing bone cell viability through the promotion of inflammation and the loss of cell protection by estrogens.

## The Gut Microbiota is Associated with Aging

The human gut microbiota comprises a complex ecosystem of bacteria, archaea, fungi and viruses that cover all parts of the digestive tract. However, even in the digestive tract, the composition and function of these consortia differ at each site, with the microbiota in the large intestine or stool being the largest and best studied. Alterations in the composition and function of the microbiota are associated with a variety of inflammatory, autoimmune, metabolic and neoplastic diseases [19]. This “dysbiosis” is usually characterized by reduced microbial diversity, the excessive growth of potentially pathogenic commensals (pathobionts), the loss of commensals or a combination of these factors [20]. However, the link between diseases and their specific microbial signatures must be interpreted with caution. While this definition emphasizes the pathological feature of dysbiosis, shifts in the microbial community may be a consequence of pathology and represent an important positive mechanism of adaptation to the diseased state. Targeting the microbes associated with dysbiosis could thus undermine this compensatory mechanism. Age-related dysbiosis has been proposed as an additional feature of aging that is mechanistically linked to chronic inflammation and immune senescence [21]. However, whether this is a loss of adaptability to changing conditions or the presence or absence of key microbes, whether this is a cause or a consequence of adaptation, or even what is low microbial diversity, remains controversial and may differ for each disease [22].

Owing to the abundance of energy, stable temperature, low oxygen content and many other factors that create a specific niche in the human gut, the vast majority of all bacteria in the typical colonic microbiota of healthy people belong to four phyla: Firmicutes, Bacteroidetes, Actinobacteria and Proteobacteria [23]. However, changes in several external factors, such as diet, have a profound impact on these consortia even in healthy individuals. Switching from a plant-based to an animal-based diet triggers a shift from Alistipes, Bilophila and Bacteroides to Roseburia, *Eubacterium rectale* and *Ruminococcus bromii* [24], and the abundance of 15% of all microbes fluctuates daily, which is simply due to the alternation between eating and fasting [25]. This shaping of the gut microbiota can influence the ability of the immune system to induce inflammation, which in turn affects bone health [26]. However, while the gut microbiota and the formation of short-chain fatty acids change with diet, multiple studies analyzing specific diets (e.g., the Mediterranean diet) often do not show clear or consistent effects on the gut microbiota [27]. This discrepancy could be due to the considerable interindividual variability in the composition of the microbiota caused by other variables, such as the age of the participants [28] or the degree of urbanization of their environment [29].

The gut microbiota changes with age. In the first 3 years of life, microbial consortia rapidly develop as the infant’s gut adapts to the environment and changes in food sources. Even if the main components of the gut microbiota are already firmly established at this time, the gut microbiota will continue to change in older people. This development is associated with other factors associated with aging, such as low-grade inflammation, immunosenescence or loss of gut barrier integrity and bone mass. In both women and men, the function of intestinal epithelial cells and gut barrier integrity deteriorate with age, changes in hormonal status, and other environmental factors [30]. The gut microbiota of older people also differs from that of younger people, but these changes occur gradually and without a chronological threshold [28]. The microbiota of older people appears to be less stable than that of younger people [31]. From the fourth decade of life onward, the relative abundance of Actinobacteria, Bifidobacteria and several members of the phylum Firmicutes with anti-inflammatory properties, including *Clostridium* cluster IV and *Clostridium* cluster XIVa, decreases [32, 33], and the abundance of Proteobacteria and Bacteroidetes increases [34–37]. The combination of probiotics and prebiotics, which leads to anti-inflammatory tuning of immunity, may help control inflammation in elderly individuals and promote healthy aging (see [38]).

In people older than 100 years, osteoporosis develops much more slowly in the oldest age group than in younger centenarians or in the control population [39]. Moreover, people older than 105 years have a greater gut microbiota diversity and different gut microbiota representations (higher representation of the genera *Akkermansia* and *Bifidobacterium* and the family *Christensenellaceae*) than does the general population of centenarians [40, 41]. These data were obtained mainly by comparing cohorts of people of different ages. This cross-sectional study design cannot distinguish whether these microbial characteristics are a cause or consequence of longevity or whether they are caused only by a specific condition at the time of colonization and are not related to longevity. *Lactobacillus amylovorus* in the gut is associated with an increased likelihood of longevity, whereas *F. nucleatum* is negatively associated with longevity [40]. However, this microbial signature is associated with decreased body adiposity [42] and a decreased risk of colorectal cancer [43], which could influence longevity. Nevertheless, these associations are still part of the emerging science and studies finding this microbial signature are criticized for their technical, methodological or design shortcomings and because only some isolates of the associated bacteria can drive the disease [44, 45].

## The Gut Microbiota is Associated with Estrogen Status

The intestinal microbiota modulates estrogen levels [46], and estrogens also influence the intestinal microbiota [47]. In humans, genes from more than 60 genera of gut bacteria, collectively referred to as the estrobolome, can metabolize estrogens. These include β-glucuronidases, which deconjugate estrogens into biologically active forms and enable their reuptake into the bloodstream [48]. However, the gut microbiota also converts polycyclic aromatic hydrocarbons into estrogenic compounds, suggesting a mechanism by which these pollutants disrupt the endocrine system [49]. The estrogen status also significantly impacts gut motility, which indirectly alters the gut microbiota community [50] and gut microbiota can influence gut motility via the release of metabolites or end products of bacterial fermentation [51]. Therefore, sex steroids alter the microbiota and the microbiota influences its metabolism, creating important communication pathways between the host and microbes. These pathways utilize hypothalamic–pituitary–adrenal activity and autonomic nervous system function. While both estrogens and androgens are influenced by the microbiota, each is impacted differently, which has important implications for sex- and age-related differences in disease susceptibility [52, 53].

The production of sex hormones is an important factor for interindividual differences in the diversity of the gut microbiota. The absence of sex hormones reduces the expression of tight junction proteins, which affects the structure of tight junctions and impairs the function of the intestinal barrier [54, 55]. Therefore, both dysbiosis and intestinal barrier failure can be accelerated by the absence of sex hormones [55, 56]. The relationship between sex hormones and the gut microbiota has been shown in a number of studies in rodents, allowing for better control of interindividual variability, and demonstrating an increase in the relative abundance of Firmicutes and Proteobacteria, with decreases in Bacteroidetes [57–61]. Ovariectomy in rats significantly reduced species richness and significantly shifted β diversity as early as 4 weeks after ovariectomy. This shift is caused by a significantly increased proportion of several bacterial species (*Helicobacter rodentium*, Lachnospiraceae 10–1, Lachnospiraceae A4 and *Lactobacillus reuteri*) and shows marked variability between individual rats. Estrogen supplementation not only counteracts the adverse conditions resulting from ovariectomy but also shifts the microbiota toward its normal composition [62]. These studies showed that the gut microbiota responds to sex hormone levels. However, the translation of these results to humans might be complicated by key differences related to the hormonal system (estrous vs. menstrual cycle), the gut (much shorter, faster passage), the diet (well controlled in laboratory rodents) and the variability of the microbiota (clean and controlled environment in laboratory animal facilities).

The results of studies comparing the fecal microbiota in different groups of estrogen-deficient women are summarized in Table 1. In summary, sex hormone levels influence several physiological states, but their influence on the gut microbiota in postmenopausal women is less clear [63, 64]. In some populations, a decrease in bone mass is associated with a particular microbial pattern in which some representatives of *Ruminococcus* are associated with low bone mineral density (BMD) (Table 1) [65, 66]. However, changes at the phylum level have not been reported in any studies [67, 68], suggesting that sex and comorbidities have a greater impact on changes in the gut microbiota than menopausal status does. Obesity appears to be one of the most important confounding factors in these studies [69], as controlling for age and obesity may fundamentally alter the patterns of microbiota associated with postmenopausal status [70].

**Table 1:** Association of taxonomic changes in the gut microbiota with sex hormone deficiency* or reduced bone mass* The differences are toward group marked with * (↑ = increased in * group; (p) = phylum, (c) = class, (o) = order, (f) = family, (g) = genus)

α diversity	Specific microbes	Group definition	Reference
No difference	↑ Firmicutes (p), ↑ *Prevotella* (g), ↑ *Bilophila* (g), ↓ Actinobacteria (p), ↓ *Parabacteroides* (g), ↓ *Lachnospira* (g), ↓ *Roseburia* (g),	Premenopausal (mean age 46 yrs) vs. postmenopausal* women (mean age 56 yrs)	[67]
↓ Shannon index, ↓ Genus richness	↑ *Bacteroidetes* (g) ↑ *Tolumonas* (g) ↓ *Firmicutes* (p) ↓*Roseburia* (g)	Premenopausal (mean age 53 yrs) vs. postmenopausal* women (mean age 54 yrs)	[71]
No difference	↑ Pasteurellaceae (f), ↑ Comamonadaceae (f), ↑ Lentisphaerae (f) ↓ Acholeplasmataceae (f), ↓ Enterococcaceae (f), ↓ Micrococcales (f), ↓ Christensenellaceae (f), ↓ *Ruminococcus sp CAG:379*, ↓ *Clostridium neonatale*	Premenopausal (mean age 42 yrs) vs. postmenopausal* women (mean age 59 yrs)	[70]
↓ Observed Species, ↓ Shannon index, *(No association between E2 and bacterial community structure)*	↑ Lactobacillales (o), ↑ Coriobacteriales (o), ↑ *Parabacteroides* (g), ↑ *Lactobacillus* (g) ↓*Bacteroides massiliensis*, ↓ *Lachnospira pectinoschiza,* ↓*Bacteroides coprocola* ↓ Blautia (g)	Postmenopausal women (mean age 58 yrs) with normal bone mass vs. women with postmenopausal osteoporosis and lower estrogen level*	[72]
Not analyzed	↑ Rikenellaceae (f) ↓ *Bacteroides* (g)	Postmenopausal women with higher* vs. lower fracture risk and lower* vs. higher BMD, respectively (mean age 63 yrs)	[73]
No difference	↑ Bacteroidetes (p), ↑ *Bacteriodes* (g), ↑ *Bifidobacterium* (g), ↑ *Megamonas* (g), ↑ *Prevotella* (g), ↑ *Dorea* (g), ↑ *Sutterella* (g), ↑ *Butyricimonas* (g) ↓ Firmicutes (p), ↓ *Faecalibacterium* (g), ↓ *Clostridium* (g), ↓ *Coprococcus* (g), ↓ *Roseburia* (g), ↓ *Ruminococcus* (g),	Women with premature ovarian insufficiency (mean age 35 yrs) * vs. healthy women (mean age 34 yrs)	[74]
No difference	↑ *Clostridium XLVa* (g), ↑ *Coprococcus* (g), ↑ *Lactobacillus* (g), ↑ *Eggerthella* (g), ↑ Parabacteroides (g), ↑ *Flavonifractor* (g) ↓ *Veillonella* (g), ↑ *Raoultella* (g)	Men and women with osteoporosis* (mean age 70 yrs) vs. controls (mean age 68 yrs)	[75]
No difference in Shannon or Simpson diversity indices	↑ *Bacteroides* (g), ↑ Betaproteobacteria (c), ↑ *Bacteroides stercoris*, ↑ *Adlercreutzia* (g), ↓ Clostridia (c), ↓ Methanobacteriaceae (f), ↓ Peptostreptococcaceae (f), ↓ *Turicibacter* (g), ↓ *Romboutsia* (g), ↓ unclassified *Coriobacteriia*	Postmenopausal women with normal bone mass (mean age 63 yrs) vs. postmenopausal women with osteoporosis (mean age 65 yrs) *	[76]
No differences in Chao1 and Shannon diversity indices	↑ *Lactobacillus* (g), ↑ *Ruminococcus* (g), ↑ *Bacteroides* (g) ↓ *Blautia* (g), ↓ *Alistipes* (g)	Meta-analysis, healthy men and women vs. people with osteoporosis*	[65]
↓ Shannon index	↑ Bacteroides sp.Ga6A1, *Prevotella marshii*, *Sutterella wadsworthensis,* ↓ *Escherichia coli*, *Veillonella seminalis*, *Parabacteroides johnsonii*, *[Clostridium] lactatifermentans*, *Oscillibacter sp.KLE1745*	Premenopausal (mean age 40 yrs) vs. postmenopausal (mean age 53 yrs) * women	[77]
↓ Shannon index, no differences in observed ASVs and Faith’s PD	E1: ↑ *Eubacterium ventriosum* group (g), ↑ Mollicutes.RF39 (f) E2: ↓ Burkholderiaceae (f), ↑ *Intestinibacter* (g), ↑ *unknown Clostridiales* (g),	Low levels* of estrone (E1) and estradiol (E2) in postmenopausal women (mean age 58 yrs)	[78]
No difference	↑ Fusobacteria (p), ↑ Bacilli (c), ↑ Erysipelotrichia (c), ↑ unidentified Clostridiales (g), ↑ *Clostridium disporicum*, ↑ *Lactobacillus salivarius* ↓ Ruminococcaceae (f), ↓ *Bacteroides eggerthii*	Postmenopausal women with normal bone mass (mean age 57 yrs) vs. postmenopausal women with postmenopausal osteoporosis (mean age 59 yrs)*	[79]
↓ Faith PD decrease after ≤ 12 months	Minimal changes, ↓ *Lactococcus lactis*	Paired samples of women (mean age 48 yrs) before vs. after ovariectomy*	[69]
↓ Observed Species, ↓ Shannon index	↓ *Alistipes* (g), ↓*Collinsella* (g), ↓ Erysipelotrichia (c), ↓ Clostridia (c)	Low levels* of estrogens in HIV + postmenopausal women (mean age 58 yrs)	[80]

## Leaky Gut and Inflammation Link the Gut Microbiota and Bone Health

Studies in both men and women have shown a significant correlation between the richness and diversity of the gut microbiota and bone mass [81, 82]. This correlation may not be solely due to low levels of sex hormones. Although estrogen deficiency drives bone loss in conventional mice, it is not sufficient to increase bone resorption or trabecular bone loss in germ-free mice [54]. Several specific microbial shifts are associated with low bone mass (Table 1), suggesting that there is a pathogenetic link between the gut microbiota and bone mass and quality.

Certain microbes and their products can improve intestinal barrier function and reduce inflammation. Oral administration of *Prevotella histicola* to ovariectomized mice increased the expression of tight junction proteins (zonula occludens-1 and occludin), prevented TNF-α overproduction, and halted bone loss induced by estrogen deficiency [83]. The use of probiotics also dampens chronic low-grade inflammation, although this effect may depend on the suitability of specific probiotics for the population [84]. However, improvements in bone metabolism and the prevention of bone loss could also be achieved by certain microbial products. Butyrate, a short-chain fatty acid, has anti-inflammatory effects by inhibiting NF-κB activation in intestinal epithelial cells [85] and by interacting with immune cells through specific receptors [86]. Owing to this immunomodulatory effect, a reduction in butyrate-producing bacteria, such as members of the Firmicutes phylum (e.g., *Roseburia spp.*, and *Faecalibacterium prausnitzii*), could lead to a deterioration of intestinal barrier function and even intestinal inflammation. By producing butyrate, members of the genus *Lachnospiraceae* (phylum Firmicutes), whose abundance is positively correlated with bone mass, improve the function of the intestinal barrier and prevent the transfer of bacterial lipopolysaccharides and chronic low-grade inflammation [87].

Metabolites from the gut microbiota establish a link between the microbiota and bone health in various ways. They promote bone growth and remodeling by inducing the production of insulin-like growth factor 1 (IGF-1) [88], although some specific microbes can promote bone growth through IGF-1 signaling via the innate immune receptor NOD2 in the gut [89]. SCFAs also induce the production of the gut hormones glucagon-like peptide-1 (GLP-1), GLP-2 and peptide YY from the enteroendocrine L cells of the gut. GLP-1 and GLP-2 promote bone formation, whereas the peptide YY promotes bone resorption [90, 91]. While the microbiota can promote both mechanisms, the composition of microbial signals and differences in microbial production dynamics play key roles in selecting the dominant mechanism [92].

Vitamin D is another pleiotropic factor that influences bone health directly [93], but also indirectly through modulation of the gut microbiota, gut barrier function, immune response, intestinal transcellular and paracellular transport, gut lumen pH, and short-chain fatty acid production [94]. Studies in mice suggest that the gut microbiota upregulates vitamin D receptor (VDR) through fermentation products. Loss of the VDR gene (by genetic manipulation in mice) or a VDR gene polymorphism in humans explains a significant part of the variability in the gut microbiota. Vitamin D also contributes to the maintenance of gut barrier integrity by ensuring adequate levels of antimicrobial peptides and mucus production and by strengthening intercellular junctions [95]. The effects of vitamin D may be further modulated by estrogen deficiency. Estradiol is positively associated with intestinal calcium absorption via an estrogen receptor [96, 97]. In addition, estrogen upregulates the expression of vitamin D receptor (VDR) in the rat duodenal mucosa and increases the responsiveness to endogenous 1,25(OH)_2_D [98]. Thus, vitamin D deficiency could lead to a leaky gut and inappropriate stimulation of the immune system, creating proinflammatory conditions. Vitamin D also regulates the adaptive immune system by suppressing Th1/Th17 cells and promoting Treg cells [99, 100], which could further exacerbate the inflammatory response in patients with vitamin D deficiency.

In summary, leaky gut and chronic low-grade inflammation could be common denominators linking dysbiosis, sex hormone loss and the aging of bone cells. Disruption of the intestinal barrier causes the immune system to enter a proinflammatory state, during which the cytokines and immune cells can spread throughout the body and affect bone mass and quality.

## Bone and Gut Microbiota After Menopause

Fluctuating sex hormone levels during the menstrual cycle as well as the maintenance of constant levels of estrogen and progesterone by oral contraceptives might impact the diversity and differences in the abundance of several bacterial taxa [101, 102]. Accelerated bone loss after menopause is difficult to separate from aging, and ovariectomy may not be the perfect model for evaluating menopause. While both ovariectomy and menopause lead to estrogen deficiency and are associated with accelerated bone loss, the dynamics of estrogen loss differ. Ovariectomy does not reflect the dynamics of immune responses during the physiological menopausal transition. In women, ovariectomy is associated with thymic hypertrophy [6], whereas physiologically, thymic involution ends by the fourth decade [5]. In older individuals, the vast majority of lymphocytes reside in gut-associated lymphoid tissues [103, 104]. Conversely, thymectomy significantly suppresses T-cell lymphopoiesis and bone resorption in mice after ovariectomy [105]. Nevertheless, ovariectomy is a model that can be used to evaluate the impact of estrogen deficiency alone on the relationship between the gut microbiota and bone mass. Estrogen deficiency after ovariectomy is associated with further increases in RANKL production, and osteoclastogenesis, prolongation of the lifespan of osteoclasts, increased osteoclastic bone resorption, and accelerated bone loss [106]. Osteocytes, B cells, and differentiated Th17 TNF-α^+^ cells enhance the effect of RANKL through the production of IL-17 and TNF-α [6] [107–111]. Decreased secretion of osteoprotegerin by osteocytes, as well as B cells and dendritic cells [112], prevents the interaction between RANKL and RANK and contributes to increased osteoclastogenesis and prolongation of the lifespan of osteoclasts [107]. Interestingly, RANKL is also expressed by epithelial cells throughout the intestine. The administration of exogenous RANKL to RANKL-deficient mice has demonstrated a critical role for RANKL in the differentiation of RANK-expressing enterocytes into microfold (M) cells, which are important in the induction of specific mucosal immune responses in Peyer’s patches [113–116]. There is an immunological connection between the bone marrow and the digestive tract. Ovariectomy in mice leads to increased gut permeability and subsequently increased bacterial translocation. Experimental studies have clearly demonstrated a link between intestinal dysbiosis and bone loss [61] and an association between intestinal barrier dysfunction in the context of gut dysbiosis and a reduction in bone mass [117]. These mechanisms potentiate one other, as the dysbiotic gut microbiota interacts more strongly with the immune system when the gut barrier is disturbed, resulting in chronic low-grade inflammation.

This inflammation leads to increased local production of IFN-γ and TNF-α, which exacerbates gut barrier dysfunction by decreasing the expression of tight junction proteins in epithelial and endothelial cells [118, 119]. TNF-α increases the number of Th17 cells (CD4^+^ IL-17A^+^ T lymphocytes) in the intestine and directs the migration of Th17 cells from the intestine to the bone marrow by upregulating CCL20 [120]. Translocated gut microbes, proinflammatory microbial components (e.g., lipopolysaccharides), cytokines and stimulated immune cells can enter the systemic circulation and induce changes in distant organs, including bone loss. The antigen-independent production of IL-17 and TNF-α by memory T cells during the loss of estrogen production is driven by the simultaneous overproduction of IL-7 and IL-15 by dendritic cells in the bone marrow [121]. IL-17 stimulates the release of RANKL from osteoblasts and osteocytes [122] and upregulates RANK expression, thereby increasing the osteoclastogenic activity of RANKL. IL-17, RANKL or TNF-α blocking antibodies prevent the deterioration of trabecular microarchitecture in ovariectomized mice [123]. By blocking the exit of T lymphocytes from the intestine into the bloodstream and their entry into the bone marrow, both the expansion of T cells in the bone marrow and trabecular bone loss are prevented [120]. Blocking T-cell migration or treatment with TNF inhibitors [108] could therefore be an alternative to prevent bone resorption in estrogen-deficient postmenopausal women. These findings show the importance of immune system activation for bone resorption in the absence of estrogen.

The dynamics of changes in bone mass and the fecal microbiota after ovariectomy in women are not as rapid as those reported in the experimental studies mentioned above. According to a prospective controlled study of women ovariectomized at their reproductive age, the fecal microbiota diversity did not change during the first six months after surgery compared to that before surgery, whereas the bone remodeling marker values increased significantly, and the lumbar spine BMD decreased significantly. The decrease in lumbar spine BMD continued over the following year, whereas the rate of bone remodeling remained elevated. However, after ovariectomy, it took 18 months for the gut microbiota to significantly change [69]. As the direct effects of estradiol deficiency on the bone marrow can be observed in women in the first months after ovariectomy, further studies should investigate whether and to what extent the migration of T lymphocytes from the intestinal lymphoid tissue contributes to bone marrow expansion.

## Menopausal Hormone Therapy and the Gut Microbiota

The presence of estrogen receptors (ERα, ERβ, and the G-protein-coupled receptor GPER) and androgen receptors in the intestinal epithelium [124] explains the effects of hormone therapy on the diversity of the gut microbiota and intestinal barrier function in experimental studies [54, 59, 125, 126]. In postmenopausal women treated with estrogens, the abundance of Proteobacteria and Bacteroidetes in the duodenum reached premenopausal levels [127]. In women, one year of daily low-dose estradiol treatment prevented the decrease in the diversity of the intestinal microbiota typical of women not receiving hormone treatment [69]. However, further studies are needed to evaluate the associations of estrogen levels with changes in the gut microbiota and the aging of intestinal cells [128–131]. As hormone therapy in women after physiological and artificial menopause [69, 127] alters the state of the gut microbiota, further studies are needed to verify the extent to which the decline in bone mass after several years in postmenopausal women is influenced by the migration of T lymphocytes from the lymphoid tissue of the gut. The differentiation of Th17 cells in humans can be triggered by two dozen nonpathogenic intestinal bacteria [132]. Blocking the migration of T lymphocytes from the intestine to the bone marrow could be a therapeutic alternative for preventing postmenopausal bone loss. An inhibitor of the sphingosine-1-phosphate receptor stops the transport of lymphocytes from Peyer's patches and mesenteric lymph nodes without affecting the function of lymphocytes and reduces the number of Th17 cells in the bone marrow [133].

## Questions, Problems, Controversies and the Way Forward

There is a constant bidirectional interaction among the gut microbiota, sex hormones and immune system and bone health can be influenced by these interactions. Key mechanisms include dysbiosis, an increase in gut permeability and chronic low-grade inflammation. However, despite a wealth of valuable and intriguing findings, the search for microbial biomarkers has not yet identified any universal microbial taxa that could be used to target or predict age-related bone loss. The gut microbiota of estrogen-deficient women is usually less diverse than that of healthy individuals and often has a similar pattern of enrichment (e.g., *A. muciniphila*) or depletion (e.g., several members of the genus *Clostridium*) across multiple studies. The significance and relevance of these changes are difficult to assess owing to the variability of study results and the presence of numerous age-related comorbidities. In addition, only some microbes of a particular species may influence disease progression [45]. Therefore, multiomics integration of the available microbial community data may be needed to identify the most important microbial factors [134].

The gut microbiota adapts to the environment, and its high diversity may represent the ability of the microbiome to react to the ever-changing world. In fact, a decrease in gut microbiota diversity may signal specialization of the gut microbiota, which could improve efficiency in the less volatile environment of the industrialized world [135]. There is still a lack of knowledge on how the function of one microbe can impact another, but it is clear that the metabolic activity of the gut microbiota is much less volatile than its taxonomic composition [136]. For example, a significant increase in estrogen levels during the third trimester of pregnancy is associated with decreased phylogenetic diversity and a significant shift in the β diversity of the gut microbiota without significant changes in the microbiome's gene repertoire [137]. Thus, studies comparing microbial metabolism may lead to faster recognition of key microbial traits than studies of microbial taxonomy, especially if there are no paired samples from prospective studies available. However, for interactions with the immune system, structural components of the microbiota are still quite important, and these structural components can differ between microbial species or even between isolates and can be missed if only metabolism is studied [138].

Most related research has focused on the microbiota in feces, which is the largest and most easily accessible source of samples. However, an important interaction between the immune system and microbes occurs in the organized lymphoid tissue of the small intestine (e.g., Peyer’s patches). Thus, minor differences in microbial composition could be important because of the lower microbial load in the small intestine. The effects of sex hormones on the small intestinal microbiota have rarely been studied, and these microbes may be underrepresented in the stool, so small changes are easy to miss. Nevertheless, the duodenal microbiota responds to the loss of estrogens during menopause and to hormone treatment after menopause [127], suggesting that this topic is interesting for future research. These interactions may even occur during the "window of opportunity" in early life, making them even more challenging to study. The immune system (and other physiological functions) develops in infancy, and changes immune system reactivity may manifest as impaired immune system regulation later in life [139]. To develop new hypotheses suitable for clinical evaluation, studies must first be conducted in gnotobiotic models and carefully implemented in humans.

The gut microbiota, the estrobolome, clearly responds to sex hormones and has the ability to influence their metabolism. However, it is difficult to assess the relative importance of estrogen levels, as these levels may be specific to the microbes colonizing a particular individual and compensated for by the organism’s own feedback mechanisms. The increased absorption of estrogens facilitated by the gut microbiota may be compensated for by increased urinary excretion, thereby allowing the estrogens saved from stool to exit the organism via urine [140, 141]. Therefore, a careful description of the estrogen balance should be considered in future studies.

The effects of menopause, ovariectomy or even pregnancy are often summarized as changes in sex hormones. However, these are independent processes associated with their own list of age-related problems or lifestyle factors. Ovariectomy is perfect for analyzing the effect of estrogens on the microbiota (and bone), but may not be the perfect model for evaluating menopause. Both lead to estrogen deficiency, and both are associated with osteoporosis, but the dynamics of estrogen loss are different. Menopause is not just the loss of estrogen; postmenopausal osteoporosis is difficult to separate from aging. The extent of the inflammatory response and gut barrier failure may influence not only bone mass, but also other symptoms of menopause [142]. Obesity, leaky gut and some diseases associated with aging are known modulators of the microbiota. Therefore, the results of these studies must be interpreted with full knowledge of these confounding factors.

## Conclusion

Primary osteoporosis is a global public health problem for individuals and society. The timely prevention of low-impact fractures is becoming increasingly urgent. Chronic low-grade inflammation, triggered by hormonal changes, leaky gut and cellular senescence, could represent an interesting therapeutic target. Suppressing inflammation appears to be a viable strategy for reducing the risk of osteoporosis. This strategy has been demonstrated for physical activity [143] and menopausal hormone therapy [144]. Estrogens suppress bone and immune cell senescence, decrease RANKL production, reduce osteoclastogenesis, prolong the lifespan of osteoclasts, decrease osteoclastic bone resorption, and attenuate bone loss. Interestingly, changes in the gut microbiota are associated with most steps in the pathogenesis of osteoporosis, making them prominent targets for novel therapeutic research. Some gut microbes are associated with an increase in the return of estrogens to the host, improved gut barrier function and dampened inflammation. However, deciphering the complex pathogenesis of osteoporosis and the mechanisms linking the gut microbiota to bone health is complicated by numerous age-related comorbidities.

## Data Availability

No datasets were generated or analysed during the current study.
